# Cognitive ability and self-control in relation to dietary habits, physical activity and bodyweight in adolescents

**DOI:** 10.1186/1479-5868-7-22

**Published:** 2010-03-23

**Authors:** Marianne Junger, Margit van Kampen

**Affiliations:** 1 Institute for Social Safety Studies, School of Management & Governance, University of Twente, PO Box 217, 7500 AE Enschede, the Netherlands; 2 Eenheid Zorg, Slaak 45, 3061 CR Rotterdam, the Netherlands

## Abstract

**Background:**

Previous studies showed that cognitive ability is related to health and mortality. The cause of this relationship remains largely unknown. One plausible explanation is that cognitive ability is related to behaviours that affect health. This study investigates whether cognitive ability is related to healthy dietary habits, physical activity and appropriate bodyweight in adolescents and examines whether self-control mediates the relationship between cognitive ability and health behaviour.

**Methods:**

In total 201 high-school students aged between 15 and 20 participated in the study. They completed three cognitive tests, measuring cognitive ability, reaction time and memory span, and completed a questionnaire on self-control, dietary habits, physical activity and bodyweight.

**Results:**

Results show that adolescents scoring high on the cognitive ability test have healthier dietary habits and engage more often in physical activity. Adolescents with high self-control have a healthier eating pattern, are more often physically active and have lower BMI's. Both reaction time and memory span were not related to dietary habits and physical activity. Self-control was not related to cognitive ability and could not, therefore, mediate the relationship between cognitive ability and health in this study.

**Conclusion:**

In conclusion, the link between cognitive ability and health behaviour could explain - in part - the relationship between cognitive ability and health. Self-control cannot explain this link.

## Background

Recent studies [1-3] found that there is a positive relationship between cognitive ability and health. Some studies focussed on reaction time and memory span as more basic capacities linked to health [4-7]. Until now, few studies have focused explicitly on explaining this relationship [1,2]. A number of possible explanations have been proposed.

Lower cognitive ability predicts poorer health outcomes in general [1-3] as well as multiple causes of mortality [1,8-15]. The Scottish Mental Surveys of 1932 and 1947 illustrate this relationship. These studies measured cognitive ability for almost everyone born in 1921 (N = 89,498) and 1936 (N = 70,805). It was found that, after adjusting for social class and deprivation, higher childhood cognitive ability at age 11 was related to less all-cause and cardiovascular mortality, and to increased smoking cessation by midlife. Subjects with a disadvantage of 1 standard deviation (15 points) in IQ-score relative to others were only 79% as likely to live up to age 76 [14].

Most studies looked at general cognitive ability in relation to health. In contrast, other studies examined the relationship between reaction time, working memory and health. Reaction time and memory span are considered to be aspects of cognitive ability or determinants of it [4,5,16-22]. Longer reaction times are related to higher mortality risk [6] and to a lower amount of physical exercise [23]. Accordingly, Deary & Der [5] proposed that reaction time instead of general cognitive ability might be the most important predictor of health and mortality. They argued that reaction time is an aspect of general cognitive ability and the mechanism that accounts for the link between mental ability and mortality. Memory span has also been found to be related to mortality among young adults [6].

The reasons for the association between cognitive ability and health are still unclear. Several explanations have been offered. A possible explanation is that cognitive ability promotes healthy behaviour [14,15,24]. Individuals with higher cognitive ability have more mental resources that may help to prevent illness and accidents. Cognitive ability is manifested in thinking skills, such as learning, reasoning and problem solving [16,25,26]. These abilities are important in complex situations. Health self-care is a changing and complex task in which one has to protect oneself from accidents and diseases. In health self-care, thinking skills might therefore play an important role [14,15,24]. Consequently, intelligent persons may develop healthier behaviour, e.g. smoke less, be moderate with alcohol use and have healthy dietary habits, all behaviours contributing to all-mortality risk [27-29]. In support of this, one study reported that cognitive ability was related to less smoking and a higher likelihood of giving up smoking. Also, intelligent people less often have overweight and obesity problems [30]. In line with these findings, the present study will investigate the relationship between cognitive ability, reaction time and memory span on the one hand and health related behaviour and body weight on the other hand.

A different body of research in the field of health related behaviour has investigated the importance of self-control. Self-control is conceived as an essential and basic mental resource: through self-control persons are able to inhibit or change their inner responses and refrain from acting out undesirable behaviours [31,32]. It refers to the ability to refrain from impulsive actions that are detrimental on the long term, and as such, it is a fundamental and major determinant of behaviour in general [31,33] and more specifically for deviant behaviour in childhood [34-36], adolescence and adulthood [37,38] and health behaviour at all ages [39-41]. It has been argued that self-control may be constitutional [42], the result of adequate parenting [38], or a combination of both [33]. Persons with high self-control show less dysfunctional, impulsive behaviours than persons with low self-control [38,40,43].

Baumeister and colleagues have proposed self-control as a major personality characteristic that explains a broad range of health related behaviours [39-41,44,45]. They showed that adolescents with high self-control have a healthier lifestyle than those with low self-control. Furthermore, high scores on self-control correlated with better school performance, measured as higher grade point average [40].

On the basis of the findings described above, it is expected that there is a relationship between self-control and health related behaviour.

Therefore, we hypothesize that self-control might explain the relationship between health related behaviour and cognitive ability: cognitive ability might promote the acquisition of self-control and self-control leads to healthy behaviour. Because cognitive ability is manifested in thinking skills, such as learning, reasoning and problem solving [16,25,26], it can be argued that cognitive ability also contributes to the acquisition of self-control. A recent meta-analysis found a modest relationship of -0.23 between Delay Discounting and cognitive ability [46]. Delay discounting measures the inability to resist the temptation of a smaller immediate reward instead of receiving a larger reward at a later date. The other way around is also plausible: it is possible that self-control helps to achieve good results on cognitive tests. One recent study - the first to investigate this relationship - found support for this thesis and reported that various personality measures, mostly but not always related to aspects of self-control (consciousness, performance motivation, fear of failure, openness) are related to cognitive performance [47].

The present study investigates three issues. First, it investigates if there is a relationship between cognitive ability, reaction time and memory span and health related behaviour. Second, it investigates whether self-control is related to health related behaviour and, third, whether self-control mediates the relationship between cognitive ability, reaction time and memory span on the one hand and health related behaviour and body weight on the other hand. Health related behaviours are operationalised as unhealthy dietary habits, and physical activity. The study is based on a sample of adolescents.

## Method

### Subjects

The sample consisted of 201 adolescents (105 women) between 15 and 20 years old, all of whom lived at home. Adolescence is an important period for the development of dietary habits.

The sample was a convenience sample, but attention was paid to maximize variance in terms of cognitive ability (IQ) by selecting subjects belonging to four different levels of education. Students from eight different schools participated. The schools were located in different cities in the south and the centre of the Netherlands (Eindhoven, Sleeuwijk, Culemborg, Arnhem, Utrecht, Huizen, Oosterhout). Around one third of the subjects was attending schools for vocational training VMBO-T, (pre-vocational secondary education; four years) or MBO (vocational secondary education; three years), one third HAVO high school (senior general secondary education: five years) and one third was attending a VWO high school (pre-university education: six years). To obtain correct representation of age and sex groups, researchers attempted to get equal numbers of each age-group and sex.

The researchers made contact with schools, via a person that was known to them. Via the headmaster or management of the high schools, teachers of the classes in the right age range were contacted and the teachers asked students to participate. Leaflets were distributed to explain the general aim of the study and what was expected from the students. Depending on the number of classes with students in the age of 15-20, sometimes one entire class participated and sometimes 5 to 6 students per class were requested to participate, in larger schools, they were asked to register (see procedure). Given this procedure, no non-response rate could be computed, but practically all students who were explicitly asked by the teachers actually participated in the study. Data collection was done during school time.

### Measures

Information on dietary habits, body weight, self-control and the control variables was obtained through a questionnaire which respondents filled in on a personal computer.

To collect information about *dietary habits*, questions were used that came from the questionnaire used in the Dutch Health Behaviour in School-aged Children (HBSC)-study on adolescent health, well-being and health-behaviour [48], which was part of a cross-national health study in collaboration with the World Health Organization [49].

- Frequency of having *breakfast *was measured with two questions that focused on week-days and week-ends: 'How often do you usually have breakfast, more than a glass of milk or juice, during the week?' (5-point format, ranging from 0 = never to 5 = 5 days) and 'How often do you usually have breakfast, more than a glass of milk or juice, during the weekend?' (0 = never, 1 = 1 day, 2 = 2 days). The reason for measuring week-days and week-ends separately was the assumption that these two questions represent somewhat different constructs. Parents might have more influence on breakfast consumption during the week-end than during the week. In support, the correlation coefficient was not high enough (r = 0.40, p < .001) to form a scale.

- Subjects were asked - in two separate questions - to rate the frequency of their consumption of *fruits *and of *vegetables *on a 7-point scale (never, less than once a week, once a week, 2-4 days a week, 5-6 days a week, once a day, more than once a day). Again, the correlation coefficient was not high enough (r = 0.33, p < .001) to form a scale.

*- Unhealthy food *was measured by asking about intake of sweets (candy or chocolate), soft drinks, snacks (e.g. fries, hamburger) and crisps on a 7-point scale (never, less than once a week, once a week, 2-4 days a week, 5-6 days a week, once a day, more than once a day). These four items formed a scale "unhealthy food" (Cronbach's α = 0.65).

Scores on the unhealthy dietary habits were recoded, so that for cognitive ability and memory span a positive correlation with dietary habits means that subjects with a higher score have healthier dietary habits.

- Subjects were also asked how much *money on average they spend daily *on snacks, namely on candy, snacks or soft drinks (answers in Euro's).

*- Physical activity *was assessed using the 60 min Moderate-to-Vigorous Physical Activity (MVPA) measure [50]. The MVPA was found to be a valid and reliable measure of physical activity. It consists of two questions: 'Over the past 7 days, how many days were you physically active for a total of at least 60 min per day?' and 'Over a typical or usual week, how many days were you physically active for a total of at least 60 min per day?' The average number of days of the past and a typical week was an index for engaging in physical activity.

*- Bodyweight *was measured with questions about subject's weight and height and converted into the Body Mass Index (BMI, kg.m^-2^).

*- Cognitive ability *was measured with the Raven Standard Progressive Matrices (RSPM; [51,52]. The RSPM measures general cognitive ability, the ability to solve problems and understand the relationship of various concepts [53]. The test demands analytic reasoning on abstract visuospatial material [54]. The RSPM consisted of 60 problems in which subjects had to find the correct item that was missing in the pattern out of 6 or 8 possible items. During the test problems became more difficult and abstract. A computerized version of the RSPM was used. The computerized version is found to be equivalent to the standard version [55]. First, subjects received 5 problems to practice after which all 60 problems were administered. The answering time was not limited. For every correct item one point was given with a maximum score of 60. The scores were not standardized for norms of age group, because age was controlled for in the analysis. The RSPM is a valid and reliable measure for cognitive ability [51,54].

- The *Simple Reaction Times *test measured the time between a circle turning green and subject's response by pushing the space button. The circle appeared each time at a different place on the screen and the time before it turned green was variable, between 500 and 2000 milliseconds. First, subjects had 10 trials to practice. Then two blocks, each consisting of 24 trials, were administered. For every trial, reaction time was measured and the mean and median reaction time was calculated for every block. A lower score means better cognitive functioning.

- The *Corsi block-tapping task *[56] measured memory span. Nine green squares appeared on the screen in asymmetrical order. At each trial a few squares turned blue in a certain sequence. In the first part of the test, subjects were asked to recall the sequence. In the second part, subjects had to recall the sequence in the contrary order. The answering time was not limited. In both parts, the number of squares was increased by one square after every two sequences. Two trials of each sequence length were presented. The shortest sequences included two squares and the longest ones nine squares. The task was ended if both trials of each sequence length were incorrect. To calculate the score, the block span was multiplied by the number of correct trials. The maximum score was 162. A higher score means better memory span.

*- Self-control *was measured with the Dutch version of the Self-Control Scale [40,57] (Cronbach's *α *= 0.89). It consists of 36 questions about self-control (e.g. "I am good at resisting temptation"). Subjects rated their self-control on a 5-point scale (anchors 1 = not at all like me, 5 = very much like me). Because the relation between self-control and health was examined, three items in the Self-Control Scale items that pertained to health ("I engage in healthy practices", "I eat healthy foods", "I sometimes drink or use drugs to excess") were removed from the scale. Negatively formulated items were reverse-scored. A higher score means a greater ability to control oneself.

Control variables were sex, age, occupation of the primary wage earner and family income. Sex was coded 0 for females and 1 for males. Occupation of the primary wage earner was measured with a 6-point format (derived from Vazsonyi, Pickering, Junger & Hessing, 2001). Six categories of professions were specified. Each category contained descriptions of sample jobs that would fit in that category. Participants had to indicate the number of the category that contained the most accurate description of the profession of the primary wage earner. The categories were: 1 = labourer or service worker, 2 = semi-skilled worker, 3 = clerical staff, 4 = semi-professional, skilled labourer, 5 = owner of a small business, professional, 6 = owner of a large business, executive. Family income was measured through three items: yearly income of participant's parents (3-point format, 1 = less than average, 2 = average, 3 = more than average), the kind of house the subject lived in (caravan, apartment or detached house) and if subject's parents rent or own the house. This method to measure income was chosen, because it was assumed that the adolescents do not have insight in the exact income of their parents.

### Procedure

In total 10 researchers were responsible for administering the tests. All tests and questionnaires were completed on a computer. In the Mental Information processing and Neuropsychological Diagnostic System (MINDS) [58] a test battery was made in which the tests automatically appeared in the following order: reaction times test, Raven's Standard Progressive Matrices, Corsi block-tapping task, Self-Control Scale and the health survey as last. Subjects could sign up or were personally asked by the experimenters or high school teachers to participate in the study. Subjects were tested in small groups ranging from 2 to 6 pupils. They were tested in a separate room or quiet part of the library in their school were they could work on the tests quietly without being interrupted or distracted and in the presence of at least one of the researchers.

Afterwards participants received something to eat and drink for participating in the study, which they didn't know prior to the test. They received a chocolate bar or a small bag of potato chips and a soft drink. Total duration of the administration was between 30 and 50 minutes for all subjects, with one exception which was of 20 minutes.

### Data analysis

First, Pearson's correlations were computed to examine whether cognitive ability is correlated with healthy diet, physical activity and bodyweight. Second, for each outcome, stepwise regression analysis were computed. In a first step the control variables were entered, in a second step cognitive ability was added and in the final step self-control was added. In a preliminary analysis, the distribution of the predictor and outcome variables was examined for deviations from normality. The variables own/rent the house, reaction time, breakfast during the week, breakfast during the weekend and money spend on unhealthy foods deviated, and therefore logistic transformations were computed for these five variables. Stepwise regression analysis computed with these transformed variables provided the same results at the analysis with the untransformed variables. Therefore only the results from the regression analysis with the untransformed variables are presented below.

## Results

Table 1 describes the statistics of all the variables in the study (means, standard deviations, minima and maxima).

**Table 1:** Descriptive statistics: control variables, predictor variables and eating pattern, physical activity and BMI.

	*M*	*SD*	Minimum	Maximum
1. Age	16.78	.98	15	20
2. Sex	.48	.50	0	1
3. Own house	1.89	.32	1	2
4. Family income	2.65	.60	1	3
5. Kind of house	2.31	.96	1	4
6. Occupation primary wage earner	4.68	1.10	1	6
7. Cognitive ability	46.37	7.01	20	60
8. Reaction time	275.89	43.51	214	659
9. Memory Span Forward	5.55	1.82	1	9
10. Memory Score Forward	49.33	26.58	2	126
11. Memory Span Backward	4.94	1.72	1	8
12. Memory Score Backward	37.29	21.19	1	88
13. Self-control	105.25	15.52	61	139
14. Breakfast week	5.39	1.36	1	6
15. Breakfast weekend	2.76	.53	1	3
16. Fruit	4.41	1.52	1	7
17. Vegetables	5.12	.93	2	7
18. Candy	3.38	1.40	1	7
19. Soft drinks	3.32	1.80	1	7
20. Crisps	4.49	1.19	1	7
21. Snacks	5.17	.90	1	7
22. Money	2.05	2.49	0	15
23. Physical activity	3.94	2.03	0	7
24. BMI	21.12	2.42	16.60	30.67

Correlational analysis (table not shown) shows that higher scores on cognitive ability (RSPM) were related to faster reaction times (r = -.31; p < .01) and longer memory span (r varying between r = .17 (p < .05) and r = .27 (p < .01)). However, reaction times and memory span were hardly related to each other, with one exception: subjects who were fast on the reaction time test had a higher score on the backward memory span (r = -.18; p < .05).

Only cognitive ability and self-control were used as independent variables, in addition to the control variables, in the hierarchal regression analysis. Almost none of the correlations between reaction time and memory span and dietary habits, physical activity and BMI were statistically significant. There was one exception: drinking soft drinks was related positively to memory span forward but negatively to memory span backward (|r| varying between .15 and .17 (p < .05). Additional analysis also showed that memory span and reaction time were not related to the dependent variables in any of the multiple regression analysis. More details can be obtained from the first author.

The results of the hierarchal regression analysis are presented below (table 2, table 3 and table 4).

**Table 2:** Hierarchal regression for control-variables, self-control and psychometric cognitive ability predicting different health behaviours

	Breakfast week			Breakfast weekend			Fruit		
			
Variables	*B*	*SE B*	*β*	*B*	*SE B*	*β*	*B*	*SE B*	*β*
Step 1: Control variables added									
Age	-.09	.10	-.06	-.07	.04	-.14	-.15	.11	-.10
Sex	.05	.20	.02	-.16	.08	-.15*	-.64	.22	-.21**
Own or rent the house	.22	.34	.05	.14	.13	.09	.02	.37	.00
Family income	.33	.19	.15	.05	.07	.06	.27	.21	.11
Kind of house	-.09	.11	-.06	-.08	.04	-.15*	-.08	.12	-.05
Occupation primary wage earner	-.07	.10	-.06	.03	.04	.07	.12	.11	.08
Step 2: Cognitive ability added									
Age	-.09	.10	-.06	-.07	.04	-.13	-.15	.11	-.10
Sex	.05	.20	.02	-.15	.08	-.15*	-.63	.22	-.21**
Own or rent house	.22	.35	.05	.11	.13	.07	.00	.37	.00
Family income	.33	.19	.15	.04	.07	.05	.26	.21	.10
Kind of house	-.09	.11	-.06	-.08	.04	-.15^a^	-.08	.12	-.05
Occupation primary wage earner	-.07	.11	-.06	.03	.04	.06	.11	.11	.08
Cognitive ability	.00	.01	.00	.01	.01	.16*	.01	.02	.03
Step 3: Self-control added									
Age	-.08	.10	-.06	-.07	.04	-.13	-.15	.11	-.10
Sex	.05	.20	.02	-.15	.07	-.14*	-.63	.22	-.21**
Own or rent the house	.32	.34	.07	.14	.13	.08	.05	.37	.01
Family income	.30	.19	.13	.03	.07	.04	.25	.21	.10
Kind of house	-.05	.11	-.04	-.07	.04	-.13	-.06	.12	-.04
Occupation primary wage earner	-.08	.10	-.06	.03	.04	.06	.11	.11	.08
Cognitive ability	.00	.01	-.01	.01	.01	.15*	.01	.02	.02
Self-control	.02	.01	.24**	.01	.00	.18**	.01	.01	.13

For breakfast week, *R*^2 ^= .03 (ns) for step 1; Δ*R*^2 ^= .00 (ns) for step 2; Δ*R*^2 ^= .06 (p < .001) for step 3. For breakfast weekend, *R*^2 ^= .07 (p < .05) for step 1; Δ*R*^2 ^= .02 (p < .05) for step 2; Δ*R*^2 ^= .03 (*p *< .01) for step 3. For fruit, *R*^2 ^= .07 (*p *< .05) for step 1; Δ*R*^2 ^= .001 (ns) for step 2; Δ*R*^2 ^= .02 (ns) for step 3.

**Table 3:** Hierarchal regression for control-variables, self-control and psychometric cognitive ability predicting different health behaviours.

	Vegetables			Unhealthy foods			Money		
			
Variables	*B*	*SB*	*β*	*B*	*SE B*	*β*	*B*	*EB*	*β*
Step 1: Control variables added									
Age	-.04	.07	-.05	-.19	.07	-.19**	.24	.17	.10
Sex	-.29	.14	-.15*	.46	.13	.24**	1.32	.35	.26**
Own or rent the house	-.02	.23	-.01	.45	.22	.15*	-.37	.59	-.05
Family income	-.11	.13	-.07	.09	.13	.05	.31	.33	.07
Kind of house	.01	.07	.01	.06	.07	.06	.39	.19	.15*
Occupation primary wage earner	.17	.07	.20**	-.12	.07	-.13	-.07	.18	-.03
Step 2: Cognitive ability added									
Age	-.04	.07	-.04	-.19	.07	-.20**	.23	.17	.09
Sex	-.27	.13	-.15*	.44	.13	.23**	1.28	.34	.26**
Own or rent house	-.09	.23	-.03	.51	.22	.17*	-.19	.59	-.02
Family income	-.13	.13	-.08	.10	.12	.07	.36	.33	.09
Kind of house	.02	.07	.02	.06	.07	.06	.37	.19	.14*
Occupation primary wage earner	.16	.07	.19*	-.11	.07	-.12	-.04	.18	-.02
Cognitive ability	.03	.01	.20**	-.02	.01	-.17**	-.06	.02	-.18**
Step 3: Self-control added									
Age	-.04	.07	-.04	-.19	.06	-.20**	.23	.17	.09
Sex	-.27	.13	-.14*	.44	.13	.23**	1.27	.34	.26**
Own or rent the house	-.06	.23	-.02	.48	.22	.16*	-.33	.58	-.04
Family income	-.14	.13	-.09	.11	.12	.07	.40	.32	.10
Kind of house	.03	.07	.03	.04	.07	.05	.31	.19	.12
Occupation primary wage earner	.16	.07	.19*	-.10	.07	-.12	-.04	.18	-.02
Cognitive ability	.03	.01	.19*	-.02	.01	-.16*	-.06	.02	-.17**
Self-control	.01	.00	.11	-.01	.00	-.11	-.03	.01	-.20**

For vegetables,*R*^2 ^= .05 (*ns*) for step 1; Δ*R*^2 ^= .04 (*p*< .01) for step 2; Δ*R*^2 ^= .01 (ns) for step 3. For unhealthy foods, *R*^2 ^= .13 (*p *< .001) for step 1; Δ*R*^2 ^= .03(*p*< .01) for step 2; Δ*R*^2 ^= .01 (ns) for step 3. For money, *R*^2 ^= .12 (*p *< .001) for step 1; Δ*R*^2 ^= .03 (*p *< .01) for step 2; Δ*R*^2 ^= .04 (*p *< .01) for step 3.

**Table 4:** Hierarchal regression for control-variables, self-control and psychometric cognitive ability predicting different health behaviours.

	Physical activity			BMI		
		
Variables	*B*	*SE B*	*β*	*B*	*SE B*	*β*
Step 1: Control variables added						
Age	-.15	.14	-.07	.35	.17	.15*
Gender	.69	.29	.17*	.61	.35	.13
Own house	.11	.50	.02	.00	.59	.00
Family income	.15	.28	.05	.19	.33	.05
Kind of house	.29	.16	.14	-.25	.19	-.10
Occupation primary wage earner	-.02	.15	-.01	.01	.18	.00
Step 2: Cognitive ability added						
Age	-.14	.14	-.07	.35	.17	.14*
Sex	.72	.29	.18*	.59	.35	.12
Own or rent house	-.01	.49	.00	.09	.60	.01
Family income	.12	.27	.04	.21	.33	.05
Kind of house	.30	.16	.14	-.26	.19	-.10
Occupation primary wage earner	-.04	.15	-.02	.03	.18	.01
Cognitive ability	.05	.02	.16*	-.03	.02	-.09
Step 3: Self-control added						
Age	-.14	.14	-.07	.34	.17	.14*
Sex	.73	.28	.18*	.59	.35	.12
Own or rent the house	.10	.49	.02	-.03	.59	.00
Family income	.08	.27	.02	.25	.33	.06
Kind of house	.34	.16	.16*	-.30	.19	-.12
Occupation primary wage earner	-.05	.15	-.03	.03	.18	.01
Cognitive ability	.04	.02	.14*	-.03	.02	-.08
Self-control	.03	.01	.20**	-.03	.01	-.17*

For physical activity, *R*^2 ^= .07 (p < .05) for step 1; Δ*R*^2 ^= .02(p < .05) for step 2; Δ*R*^2 ^= .04 (p < .05) for step 3.

For BMI, *R*^2 ^= .05 (p > .05) for step 1; Δ*R*^2 ^= .01 (ns) for step 2; Δ*R*^2 ^= .03 (*p*< .05) for step 3.  * p < .05; ** p < .01.   ^a ^p = .06

### Dietary habits

Cognitive ability scores were positively related to having breakfast during the weekend (β = .15) and vegetable intake (β = .19), and negatively related to unhealthy food intake (β = -.16) and money spend on unhealthy foods and drinks (β = -.17). Self-control was positively correlated with having breakfast during the week (β = .24) and weekend (β = .18), and negatively correlated with money spend on unhealthy foods and drinks (β = -.20). It was marginally positively related to consumption of fruit (β = .13; p = .08).

### Physical activity

Both cognitive ability (β = .14) and self-control (β = .21) were related positively with physical activity.

### Bodyweight

No significant correlations were found between BMI-scores and cognitive ability (table 1). Follow-up analysis checked for a non-linear relationship. To this end, RSPM-scores were divided into 5 groups according to deviation from the mean: scores between mean and mean + one standard deviation (SD), scores between mean + one *SD *and mean + two *SD*, scores between mean and mean - one *SD*, scores between mean - one *SD *and mean - two *SD *and scores lower than mean - two *SD*. The BMI-scores of these five groups were compared using between group one-way Analysis of Variance (ANOVA). No significant differences were found between the groups (*F*(4,195) < 1.0). A similar additional analysis was performed to investigate whether there was a non-linear relationship between self-control and BMI and, again, this was not the case. The regression analysis showed that the control variables and cognitive ability were not related to BMI. Self-control on the other hand was negatively related (β = -.17) to BMI, so adolescents with more self-control had lower BMI's.

### Self-control as a mediator

Self-control did not correlate with cognitive ability, reaction time or memory span. In the bivariate analysis, Pearson correlations were |.12| or lower (non significant). A multivariate step-wise analysis was also performed: in a first step the socio-economic variables (the same as in the analysis presented in table 2, table 3 and table 4) were entered, in a second step, cognitive ability, memory span and reaction time were entered. This second step was not significantly related to self-control (F change = 2.1; df = 3; p = .10).

The results indicate that self-control could not mediate the relationship between cognitive ability and health behaviour in the present study and further mediation analysis was unnecessary.

## Discussion

Previous research has found that cognitive ability is related to health and mortality, but not much is known about why this relationship exists [15]. Various authors suggested that cognitive ability is related to behaviours that affect health [14,24,59]. This study was set up to examine this possibility. Three main issues were studied. First, it was investigated whether there was a relationship between cognitive ability, reaction time and memory span and health related behaviour and body weight; second, it examined whether self-control was related to health related behaviour and body weight and, third, whether self-control mediated the relationship between cognitive ability, reaction time and memory span on the one hand and health related behaviour and body weight on the other hand.

The study was based on a sample of 201 adolescents. During adolescence, individuals become more independent and more often decide themselves about their dietary habits. The consolidation of health behaviours, such as food intake and the amount of exercise, starts in childhood and early adolescence and is relatively stable thereafter [60-62]. Despite the importance of healthy nutrition and exercise in adolescence, research often shows that relatively large groups of adolescents are involved in risky health related behaviours [63].

The sample seems to be a good representation of Dutch adolescents. The occupation of the primary wage earner is similar to a sample of the general population of the same age: the mean in the present sample is 4.7 (SD = 1.1), in the sample of the general population, this was 4.6 (SD = 1.04) [64]. The scores of the RSPM in the present sample were comparable to what might be expected based on other samples (present sample: mean: 46.37 (SD: 7.01). In Ravens norms the scores for fifteen year olds were 47 (SD not mentioned) (Raven, 2000). The interrelationships between the three measures of cognitive ability were also comparable to those found in previous studies [22]. These findings indicate that the present sample is a good representation of a general sample of Dutch adolescents, and very similar to samples studied abroad.

As hypothesized, it was found that cognitive ability was positively related to health related behaviours. Adolescents with a high score on general cognitive ability had healthier dietary habits, they more often had breakfast during the weekend, ate more vegetables and less unhealthy foods, and they engaged more often in physical activity. They also spend less money on unhealthy foods and drinks (candy, snacks and soft drinks). To the best of our knowledge, no previous studies investigated these relationships.

These findings are in line with expectations formulated by several researchers in the field who suggested that the association between cognitive ability and health is mediated by the impact of cognitive ability on health related behaviours which, in turn, affects health and mortality [1-3,7-15,65].

In contrast, no relationships were found between reaction time and health related behaviour or BMI. This stands in contrast to previous, but scarce, research that reported a relationship between reaction time and higher mortality risk [6] and lower levels of physical exercise [23]. Similarly, memory span was not related to health related behaviour. Again, this contrasts with what was expected, as two previous studies found a relationship between working memory, cognitive ability and mortality [6,66]. These findings do not support Deary & Der [5]'s hypothesis that agues that reaction time instead of general cognitive ability might be the most important predictor of health and mortality. A possible explanation is that reaction time and memory span are related to health related behaviours that are different from the specific health related behaviours investigated in this study.

Self-control was also positively related to healthy eating patterns (higher frequency of having breakfast during week and weekend and lower intake of crisps and snacks) and physical activity. Adolescents with high self-control also spend less money on unhealthy foods and drinks and have lower BMI's. These findings replicate what has been found in previous research [39-41,44,57]. These findings also are in line with studies reporting a relationship between conscientiousness and longevity as the concept of conscientiousness, meaning '..self-discipline, carefulness and thoroughness' [67], is similar to that of self-control [68,69].

The analysis showed that the relationship between self-control and health behaviour and the relationship between cognitive ability and health behaviour are approximately of equal strength with β 's varying for cognitive ability between β = .14 and β = .19 and for self-control between β = .17 and β = .24.

Although in this study cognitive ability and self-control are both positively related to health behaviours, no relationship was found between cognitive ability and self-control. Therefore, self-control does not explain the relation between cognitive ability and health related behaviour in the present study. These findings suggest that self-control and cognitive ability are both independent predictors of health related behaviour.

Controlling for the variables age, gender and family income made no difference for the significance of the results. Interestingly, when controlled for family income, the amount of money spend on unhealthy foods is still related to cognitive ability and self-control. That is, less intelligent adolescents and adolescents with less self-control spend more money on unhealthy foods and drinks independent of family income.

A relation between cognitive ability and BMI was expected, since former studies found that more intelligent people are less likely to be overweight and have problems with obesity [30,70]. No such relation was found in the present study. Explanation for the lack of a relation may relate to the age of the participants and the validity of BMI development in adolescents.

There was no relationship between self-control and cognitive ability. Previous findings describe a relationship between self-control and school performance [40] and therefore a relationship between self-control and cognitive ability was hypothesized. A recent meta-analysis found a mild relationship between self-control and cognitive ability: r = -23 [46]. However, in this meta-analysis the age range was very large, from mean age 4 to 45.18. Furthermore, although the researchers controlled for age, this may have influenced the findings, as age is strongly related to self-control as well as cognitive ability scores [46]. Further, in the meta-analysis, self-control was measured through tests of delay aversion and not by a questionnaire as was the case in the present study, and this may also explain the difference in the findings. Although self-reports are amenable to biases, it is also possible that self-control as measured by tests measures to some extent 'general performance on tests' and, also, delay aversion. Subjects with relatively high cognitive ability may perform better on delay aversion tasks (show more delay), while not showing more behavioural control in real life.

The present study did not control for school type of the subjects as a possible confounder of cognitive ability. This was based on the fact that educational achievement and cognitive ability are strongly related to each other so that they are, to some extent, measures of the same construct. Several studies showed that 'ability and schooling are so strongly dependent that it is not possible, over a wide range of variation in schooling and ability, to independently vary these two variables and estimate their separate impacts' [[71], p. 1] and that 'ability and schooling effects appear to be inseparable' [[71], p. 11].

This is especially true in the Netherlands. In the Netherlands, at the end of the primary school, all children are tested, usually with the 'CITO-test'. The CITO-test is a compulsory national test for cognitive achievement comparable to the SAT, but age adjusted [72] [For an introduction of the Dutch educational system for Americans: see http://www.fulbright.nl/cache/30/30dbd0481349e7a6188e372c5b049e51/31dutchsecondaryeducation.pdf] The results of the CITO-test are used by primary schools in order to advise parents as to the type of secondary education most suited to their child [73]. This means that the school type chosen by Dutch children after primary education is heavily influenced by their cognitive abilities as measured through the CITO-test.

This system has several consequences. In a Dutch study, a relationship was found between educational achievement as measured by the CITO-test and cognitive ability of .63 at age 12. In the present study school type and cognitive ability are strongly correlated: r = .54 (p < .001). This means that controlling for school type when analyzing the relationship between cognitive ability and health related behaviour is almost the same as controlling for cognitive ability.

In an additional analysis, the regression analysis (presented in table 2, table 3 and table 4) was repeated and, in a fourth step, three dummy variables were added that coded for school type [We added 3 dummies coding for VMBO, MBO, HAVO, VWO was the reference category.]. It appeared that in seven of the eight outcome measures, the fourth step was not statistically significant. Only for 'breakfast during weekends', the fourth step was statistically significant (with p set on .05). In this case, the measure of cognitive ability (RSPM) became non significant and results show that subjects in the MBO school type have breakfast less often during weekends. It is concluded that even after controlling for school type, the findings of this study do not change.

The present study was subjected to limitations. First, the data on self-control and health behaviours was based on self-reports. The use of self-reports may have affected the results because of misinterpretation of the questions and social desirability. Findings should be replicated using other measures of self-control. Second, more intelligent participants might be better educated about healthy foods. Therefore, the results of the questionnaire might not only be representing actual health-related behaviours, but could be influenced by knowledge about healthy eating. Third, health behaviour as well as cognitive ability follows a socioeconomic gradient [74]. Although this study controlled for socioeconomic status, other factors such as parents' dietary patterns have an influence on health behaviour. Fourth, malnutrition during childhood can affect intellectual development [75]. Fifth, we used a relatively small sample. Last, because of the cross-sectional nature of the design, no causal implications can be drawn.

## Conclusions

This study is - to the best of our knowledge - the first to provide knowledge on the relationship between cognitive ability and health. On the basis of the present data it is concluded that the relationship between cognitive ability and health can at least be partly explained by the link between cognitive ability and health related behaviour. In this study, self-control does not explain the link between cognitive ability and health related behaviour. Future studies need to replicate the present findings and investigate, with different methodologies, if similar findings are found for different health behaviours as well as for other methods of measuring self-control.

Findings in this study are interesting for improving the effectiveness of health-education campaigns. Considering that less intelligent adolescents have an unhealthier diet and engage less in physical activity than intelligent adolescents, extra support and information about the importance of healthy dietary habits and exercise could be given at lower level high-schools and should be made available in understandable formats.

## Competing interests

The authors declare that they have no competing interests.

## Authors' contributions

MJ was responsible for the study, planned the study design, performed part of the statistical analysis, worked on the interpretation of the data and revised the manuscript. MvK participated in the design of the study and the data collection, performed some of the statistical analysis, helped with the interpretation of the data-analysis and drafted the first version of the manuscript. Both authors read and approved the final manuscript.
